# Process evaluation of a workplace-based health promotion and exercise cluster-randomised trial to increase productivity and reduce neck pain in office workers: a RE-AIM approach

**DOI:** 10.1186/s12889-020-8208-9

**Published:** 2020-02-04

**Authors:** Alyssa Welch, Genevieve Healy, Leon Straker, Tracy Comans, Shaun O’Leary, Markus Melloh, Gisela Sjøgaard, Michelle Pereira, Xiaoqi Chen, Venerina Johnston

**Affiliations:** 1 School of Public Health, The University of Queensland, Brisbane, 4072 Australia; 2 Centre for Health Services Research, The University of Queensland, Brisbane, 4072 Australia; 3 School of Physiotherapy and Exercise Science, Curtin University, Perth, 6845 Australia; 4 Baker Heart & Diabetes Institute, Melbourne, Victoria Australia; 5 School of Health and Rehabilitation Sciences, The University of Queensland, Brisbane, 4072 Australia; 6 Department of Physiotherapy, Royal Brisbane and Women’s Hospital, Queensland Health, Brisbane, 4029 Australia; 7 Institute of Health Sciences, Zurich University of Applied Sciences, 8401 Winterthur, Switzerland; 8 UWA Medical School, The University of Western Australia, Perth, 6009 Australia; 9 Curtin Medical School, Curtin University, Perth, 6845 Australia; 10 Department of Sport Sciences and Clinical Biomechanics, Faculty of Health Sciences, University of Southern Denmark, 5230 Odense, Denmark; 11 Health Services and Outcomes Research, National Healthcare Group, Singapore, 138543 Singapore; 12 RECOVER Injury Research Centre, The University of Queensland, Brisbane, 4072 Australia

**Keywords:** Musculoskeletal diseases, Occupational health, Workplace, Effectiveness, Evaluation

## Abstract

**Background:**

This study uses the RE-AIM framework to provide a process evaluation of a workplace-based cluster randomised trial comparing an ergonomic plus exercise intervention to an ergonomic plus health promotion intervention; and to highlight variations across organisations; and consider the implications of the findings for intervention translation.

**Method:**

This study applied the RE-AIM (reach, effectiveness, adoption, implementation, maintenance) methodology to examine the interventions’ implementation and to explore the extent to which differences between participating organisations contributed to the variations in findings. Qualitative and quantitative data collected from individual participants, research team observations and organisations were interrogated to report on the five RE-AIM domains.

**Results:**

Overall reach was 22.7% but varied across organisations (range 9 to 83%). Participants were generally representative of the recruitment pool though more females (*n* = 452 or 59%) were recruited than were in the pool (49%). Effectiveness measures (health-related productivity loss and neck pain) varied across all organisations, with no clear pattern emerging to indicate the source of the variation. Organisation-level adoption (66%) and staffing level adoption (91%) were high. The interventions were implemented with minimal protocol variations and high staffing consistency, but organisations varied in their provision of resources (e.g. training space, seniority of liaisons). Mean adherence of participants to the EET intervention was 56% during the intervention period, but varied from 41 to 71% across organisations. At 12 months, 15% of participants reported regular EET adherence. Overall mean (SD) adherence to EHP was 56% (29%) across organisations during the intervention period (range 28 to 77%), with 62% of participants reporting regular adherence at 12 months. No organisations continued the interventions after the follow-up period.

**Conclusion:**

Although the study protocol was implemented with high consistency and fidelity, variations in four domains (reach, effectiveness, adoption and implementation) arose between the 14 participating organisations. These variations may be the source of mixed effectiveness across organisations. Factors known to increase the success of workplace interventions, such as strong management support, a visible commitment to employee wellbeing and participant engagement in intervention design should be considered and adequately measured for future interventions.

**Trial registration:**

ACTRN12612001154897; 29 October 2012.

## Background

Neck pain is a major burden to industry in terms of lost productivity (reduced work performance and lost days) [1–3] and personal suffering (pain, disability, reduction in quality of life and reduced job satisfaction) [4, 5]. With more than 50% of office workers experiencing neck pain at some stage of their working life [6–8], significant resources have been allocated to prevent the onset of this problem and/or reduce the impact for the employee and employer. Strategies for the prevention and management of neck pain in office workers tend to fall into two broad categories – those targeting the individual, such as exercise training interventions; or those targeting the work environment, such as ergonomics optimisation. To understand the potential combined benefit of an ergonomic *plus* exercise intervention, a cluster randomised trial was recently conducted [9]. This trial compared a best-practice workplace based ergonomics intervention plus exercise training (EET) with a best-practice workplace based ergonomics intervention plus health promotion (EHP), on productivity and the prevention and reduction of neck pain in a population of Australian office personnel.

The primary (productivity improvements) outcomes [10] have previously been reported and the secondary outcomes (reductions in neck pain) will soon be published. The productivity analysis, conducted on an intention-to-treat basis, showed that the monetised value of health-related productivity loss was lower for the EET group than the EHP group at 12 months [10] (i.e. there was more benefit for those in the EET group than the EHP group). The analysis of all participants and a sub-analysis of those with neck pain, conducted on both an intention-to-treat and a per-protocol basis, demonstrated reductions in neck pain at 12 weeks and six months, which was maintained at 12 months for those with neck pain. No between-group differences were found, indicating that both interventions effectively reduced neck pain.

While reporting of such effectiveness outcomes is essential, so too is a comprehensive process evaluation of the intervention, as it provides context to the research findings and identifies barriers and enablers for translation of research into practice [11]. The RE-AIM framework [12, 13], with its five dimensions of reach, effectiveness, adoption, implementation and maintenance, supports such an evaluation. This framework has now been applied across multiple different interventions, including those with physical activity components [13]. It is designed to provide a framework for evaluating interventions and identifying issues that may affect dissemination and generalisation of results.

Using both qualitative and quantitative data, the aims of this study were to: provide a process evaluation of the trial using the RE-AIM framework; highlight variations across organisations; and consider the implications of the findings for intervention translation.

## Methods

### Implementation design

A prospective cluster randomised trial comparing a best practice EET with a EHP intervention was conducted in Brisbane, Australia from 2013 to 2016 (Australian New Zealand Clinical Trials Registry registration number: ACTRN12612001154897) [9]. Ethics approval was obtained from The University of Queensland Human Research Ethics Committee (# 2012001318) prior to commencement.

### Recruitment and organisational engagement

Potential organisations were identified through established industry networks, the Queensland Government workplace health and safety regulator and the professional contact networks of the research team. Inclusion criteria were: more than 50 employees; based in Brisbane; centrally located administrative staff; facilities available to support research activities; availability of an onsite liaison to coordinate activities; signed authorisation from a member of the senior leadership team; and a mix of public and private organisations.

The invitation to participate was issued to all employees (those with and without neck pain) via email through the onsite liaison with a link to an online eligibility survey. Recruitment generally occurred over a 2–3 week period. Participants were considered eligible if they were aged over 18 years and worked 30 or more hours/week performing office work. Exclusion criteria were pregnancy, health conditions such as previous trauma or injuries to the neck, specific pathologies (e.g. congenital cervical abnormalities, stenosis, radiculopathy) or inflammatory conditions (e.g. rheumatoid arthritis), any history of cervical spine surgery or if exercise was contraindicated by their medical practitioner for any reason (e.g. uncontrolled hypertension, angina) [9].

Eligible participants, who gave their consent, were clustered according to a hierarchy of organisation, building, floor, and work unit. The project coordinator assigned each participant to a cluster based on location and work unit information until the desired number of clusters was reached. In total, 100 clusters were formed. Once clusters had been formed, allocation was requested via email from a statistician blind to the identity of both the organisation and participants. Cluster allocations were requested in blocks of four (to prevent prediction of randomisation) and clusters were assigned an allocation in sequential order with 50 EET and 50 EHP clusters ultimately allocated and even clusters of EET and EHP in each organisation.

### Intervention delivery

All eligible participants received a comprehensive individual assessment of their workstation and intervention as required. Where needed, additional equipment (e.g. different chair) was either sourced onsite or purchased through the research funds.

Participants were assigned activities for one hour per week for 12 weeks (the intervention period). Details on the interventions are available from the published protocol paper [9]. In brief, the EET group received strength training for 20 min, three days each week (one supervised, two unsupervised) for 12 weeks, while the EHP group received a one hour facilitated health promotion information session each week for 12 weeks. Guidelines for the delivery of the exercise intervention were developed with associated photographs and videos and training provided to the intervention physiotherapist. All activities for both groups during the intervention period were conducted on site (in most cases in the same building) and during work hours (a pre-condition for organisational participation). Leader boards (showing teams within the organisation with the highest observed adherence for both interventions) were distributed directly to participants in each organisation every four weeks during the intervention period. EHP participants were asked to continue healthier lifestyle changes and EET participants were given exercise resistance bands and a two-week repeating program and were asked to continue exercise training on completion of the 12-week intervention with monthly follow-up reminders and data collection until 12 months post-commencement (the maintenance period).

### Data collection

All eligible consenting participants completed an online baseline survey, had their workstation assessed (with additional furniture provided as appropriate), and had physical measures (neck range of motion, neck and shoulder muscle strength and endurance measures) [9, 14] collected. These data were collected prior to intervention allocation.

The online surveys and physical measures were repeated at week 12 (end of active interventions) and 12 months post-commencement (to assess maintenance). In addition, feedback was collected from participants in the week 12 survey on overall program satisfaction and what they did and did not like about the study. Adherence to the supervised EET sessions and to EHP sessions was recorded by the session facilitators during the intervention period with online adherence surveys issued monthly (from month 4 to 11 post-commencement) during the maintenance period. EET participants were also asked to record their training in a paper-based exercise diary during the intervention period.

**Fig. 1:**
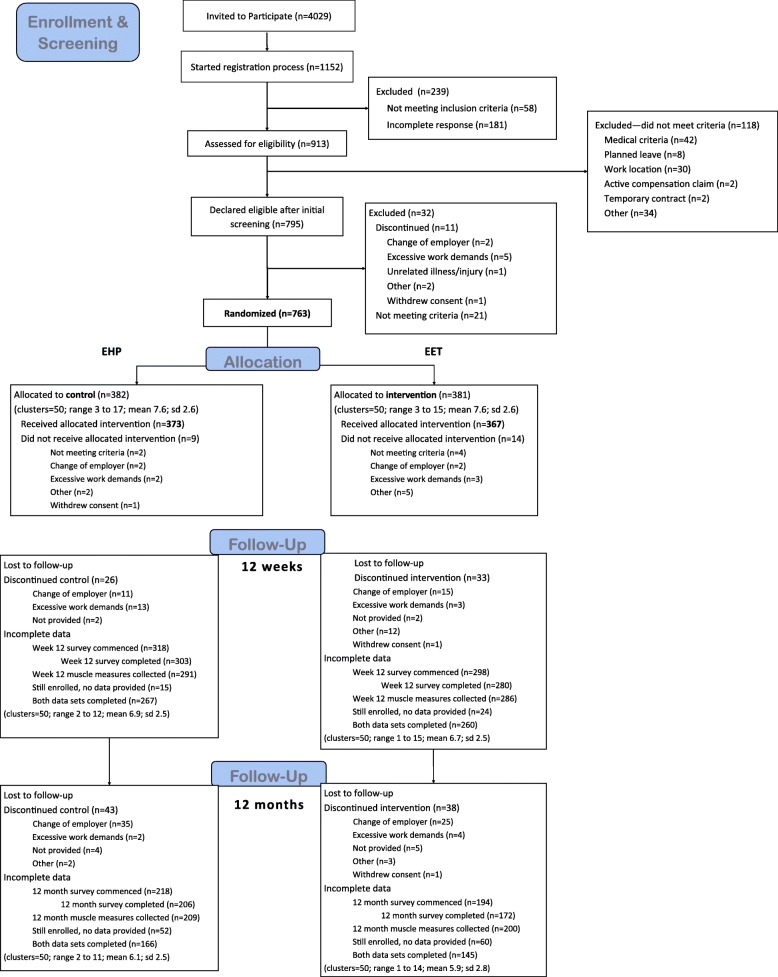
CONSORT Flow

Organisation-level data on the mean age, gender, location and income distribution by gender of all administrative staff working 30 h or more per week in the areas where recruitment occurred was provided by liaisons at each organisation. Data on eligibility screening, randomisation, implementation and maintenance can be found in the Consort Flow (Fig. 1**)**.

The research team (AW, VJ), conducted face-to-face interviews with organisational liaisons and focus groups with a sample of intervention participants from four organisations. Honest communication was encouraged to help better understand how the research worked and what could be improved for further roll-out to industry.

### Measures and analysis

A combination of the qualitative and quantitative data drawn from physical measures, surveys and interviews were used to determine the reach, effectiveness, adoption, implementation and maintenance of the workplace-based intervention. Definitions and data collection in relation to these RE-AIM criteria is summarised in Table 1.

**Table 1:** RE-AIM Criteria, definitions and data sources

Criteria [13]	Data source
**Reach**	
**Definition:** the number, proportion and representativeness (age, gender, income) of individuals who registered their interest in participating in the study and were still enrolled at the point of randomisation, compared to those who were invited to participate (recruitment pool).	
1. Exclusion criteria (% excluded or characteristics)	Study protocol Consort Flow
2. Percentage of individuals who participate, based on valid denominator	Registration survey Eligibility screening Organisational data
3. Characteristics of participants compared with nonparticipants; to local sample	Organisational data Baseline survey
4. Use of qualitative methods to understand recruitment	Week 12 survey feedback Participant focus groups
**Effectiveness**	
**Definition:** the impact of the intervention on primary (health-related productivity loss) and secondary (neck pain) outcomes, as well as other outcome measures collected.	
5. Measure of primary outcome **Productivity**: health-related productivity loss expressed in days (per 28 days). **Neck pain**: self-reported neck pain in the past 7 days on a scale of 0–9.	Participant surveys • Health and Productivity Questionnaire [15] • Neck pain [16]
6. Measure of primary outcome relative to public health goal	n/a
7. Measure of broader outcomes or use of multiple criteria (e.g. measure of quality of life or potential negative outcome)	To be reported separately
8. Measure of robustness across subgroups (e.g. moderation analyses)	Participant surveys • Health and Productivity Questionnaire [15] • Neck pain [16]
9. Measure of short-term attrition (%) and differential rates by patient characteristics or treatment group	Participant tracking data Email correspondence Baseline survey data Monthly survey data
10. Use of qualitative methods/data to understand outcomes	Week 12 survey feedback Participant focus groups
**Adoption—setting level**	
**Definition:** the absolute number, proportion and representativeness of organisations that committed to participation in the study compared to those who were approached and did not participate	
11. Setting exclusions (% or reasons or both)	Study protocol
12. Percentage of settings approached that participate (valid denominator)	Email correspondence Gatekeeper approval letters
13. Characteristics of settings participating (both comparison and intervention) compared with either [1] nonparticipants or [2] some relevant resource data	Email correspondence
14. Use of qualitative methods to understand setting level adoption	Liaison interviews
**Adoption—staff level**	
**Definition:** the absolute number, proportion and representativeness of intervention agents (research staff) that committed to participation in the study compared to those who were approached and did not participate	
15. Staff exclusions (% or reasons or both)	Management data
16. Percent of staff offered that participate	Management data
17. Characteristics of staff participants vs nonparticipating staff or typical staff	Management data
18. Use of qualitative methods to understand staff participation/staff level adoption	n/a
**Implementation**	
**Definition:** the extent to which the intervention was implemented in accordance with the study protocol [9], as well as its consistency across organisations and over the intervention period. Implementation was examined from three perspectives: the research team; the participants; and the participating organisations.	
19. Percent of perfect delivery or calls completed (e.g., fidelity) **Study-specific definitions:** Participant adherence to EET and EHP sessions during the intervention and maintenance period using “predicted total adherence” [10] to supervised and unsupervised training sessions during the intervention period and online survey questions from 12 weeks to 12 months Organisational compliance with communication strategy and provision of suitable, consistent space	Online surveys: Adherence question: “How often have you participated in the exercise training during the last 4 weeks?” (EET participants), or “How often have you practiced healthier lifestyle changes during the last 4 weeks?” (EHP participants). Participants were grouped into three categories: regular adherence (at least once a week), irregular adherence (at least twice a month), and no adherence. Workstation assessments Session facilitator records Exercise training diaries Email correspondence Project coordinator records
20. Adaptations made to intervention during study (not fidelity)	Project coordinator records
21. Cost of intervention—time	Study protocol Project coordinator records (all costs adjusted using the relevant consumer price index (CPI) category [17, 18] to June 2015, the date of the last intake.)
22. Cost of intervention—money **Study-specific definitions:** Costs calculated from an employer’s perspective	Project coordinator records Baseline surveys (salary costs)
23. Consistency of implementation across staff/time/settings/subgroups (not about differential outcomes, but process)	Session facilitator records Exercise training diaries Email correspondence Project coordinator records Online surveys of age, gender, body mass index, health-related quality of life [19], education level, occupational category, income, computer use, health, neck pain [16], job strain [20], exercise stage of change [21], exercise self-efficacy [22], psychological distress [23], physical activity levels [24], or workstation quality.
24. Use of qualitative methods to understand implementation	Week 12 survey feedback Participant focus groups
**Maintenance—individual level**	The study’s primary outcomes were reported at week 12 and 12 months. No data collection occurred after 12 months, so individual-level maintenance could not be reported (criteria 25–30)
**Maintenance—setting level**	
**Definition:** the extent to which intervention components were implemented in participating organisations after the study period. Interviews were conducted with onsite liaisons from four organisations to understand the factors affecting maintenance	
31. If program is still ongoing at 6 months post-treatment follow-up	Liaison interviews Project manager records Email correspondence
32. If and how program was adapted long-term (which elements retained after program completed)	Liaison interviews Project manager records Email correspondence
33. Some measure/discussion of alignment to organization mission or sustainability of business model	Liaison interviews Email correspondence
34. Use of qualitative methods data to understand setting level institutionalization	Liaison interviews

All statistical data were analysed using Stata/SE 15.0 (StataCorp LLC). Participants in each intervention arm have been previously determined to be comparable [10]. Intervention effectiveness in relation to productivity and pain was measured for each organisations using multi-level (individual and cluster) mixed-effects regression. Due to sample size, these models were adjusted only for the interaction of allocation and time, with unstructured covariance. The dependent variable for productivity was health-related productivity loss – the combined cost of presenteeism (being present at work without being fully productive) and health-related absenteeism, expressed in days (per 28 days) [10]. The variable for neck pain was self-reported neck pain in the past 7 days on a scale of 0–9. Independent t-tests were conducted at the organisational level to detect differences between the baseline productivity and pain scores of participants who did and did not submit data at 12 weeks and at 12 months.

Four interviews were conducted with five onsite liaisons from four organisations (Orgs 4, 7, 10 and 14). Invitations were sent to 37 participants across the same four organisations to take part in focus groups. There were insufficient available participants in Org3 and Org10, predominantly due to conflicting work demands, for the focus groups to proceed; hence, only focus group data from Orgs 4 and 14 are available. Interviews and focus groups were audio-recorded and transcribed verbatim. Free text responses to the week 12 survey were exported to Microsoft Excel. Thematic analysis was undertaken using a semantic approach to identify issues relevant to the process of the study. A coding framework based on the RE-AIM dimensions was developed a priori, with additional themes added as required during the analysis process. All texts were reviewed and coded by a single reviewer (AW) and themes were discussed with other authors (VJ and GH) and reviewed against existing literature to identify and remove any potential bias.

## Results

### Reach

Organisations were recruited across 16 intakes between 2013 and 2016. Figure 1 shows that 22.7% (*n* = 913) of the pool of 4029 employees registered their interest in participating. After being screened for eligibility, 118 people across 100 clusters were deemed to meet the exclusion criteria (details in Fig. 1) and 763 (18.9%) were randomly allocated by cluster to either EET (*n* = 381; 50 clusters) or EHP (*n* = 382; 50 clusters).

The recruitment pool was approached through the onsite liaison in each organisation, often consisted of one or more departments of larger organisations, and varied in size from 54 to 702 employees, with a median of 264.

The representativeness of potential and allocated participants based on gender, age and income is detailed in the Additional file 1: Table S1. Reach varied widely across the 14 participating organisations (from 9.4% in Org12 to 83.3% in Org14; SD 22.7%). The proportion of females recruited was higher than that in the pool (59% (*n* = 452) compared to 48.9%, respectively); however, participants were otherwise considered to be representative of the pool from which they were recruited. The percentage of participants in management positions (occupational category manager or senior official) varied between organisations. Across all organisations, 19.2% of participants were managers, but this ranged from 4% (in Org4) to 32.5% (in Org14).

When asked during participant focus groups about their reasons for participating, the key themes identified were; health-related (e.g. “*need to do something to get healthier, and usually when you come to work, you just sort of get into the job and don’t go for walks. So, it was an opportunity to … see whether by participating in the program I’d actually get moving a bit more”* (female participant, Org 14); because it was supported by management (e.g. “*they made it clear that it was pre-approved and you didn’t need to talk to your manager, you can, you can just do it”* (female participant, Org14); and the convenience of the intervention (e.g. “*you didn’t have to go and get changed or do anything out of the ordinary, it was just go in your work clothes.”* male participant, Org14).

### Effectiveness

The size and significance of the intervention effects varied between organisations (Table 3). The study was not powered to detect changes at the organisational level, and t-tests revealed significant differences in some organisations between the baseline productivity and pain scores of participants who did and did not submit data at 12 weeks and 12 months (reported in the Supplement, Additional file 1:Table S2). In brief, participants completing the week 12 and 12 month surveys in some organisations had significantly higher or lower baseline productivity loss than those who didn’t complete the surveys, while baseline neck pain did not differ between participants who did and did not submit survey data, with the exception of Org14, where people who submitted week 12 data reported significantly higher neck pain (2.1) at baseline than those who did not. Consequently, the results presented here should be treated with caution. For productivity loss, results are reported here as cost in days (per 28 days) rather than the monetised cost due to variations in mean participant income between organisations. The change to cost in days (per 28 day period) to each organisation of both sickness absenteeism and presenteeism (at work, but performing at a reduced capacity) ranged from − 0.2 (Org9) to 0.4 days (Org2, Org3 and Org11) for all participants at 12 weeks (− 0.3 (Org7 and Org11) to 0.4 days (Org4) for EET participants); and from − 0.2 days (Org4) to 0.5 days (Org2 and Org3) for all participants at 12 months (− 0.4 (Org7) to 0.4 days (Org4) for EET participants. The organisations with the most notable changes were Org2 and Org3, which reported the highest increase in health-related productivity loss for all participants at both 12 weeks and 12 months, while the cost decreased for EET participants at both time points (− 0.1 and − 0.2 respectively).

For neck pain, the change in self-reported neck pain in the previous 7 days (rated from 0 to 9) in each organisation ranged from − 1.0 (Org1) to 0.9 (Org12) out of nine for all participants at 12 weeks (− 1.4 (Org12) to 0.6 (Org6) for EET participants); and from − 1.0 (Org1 and Org14) to 1.0 (Org5) for all participants at 12 months (− 1.7 (Org5) to 0.8 (Org1) for EET participants). The organisation with the most notable changes in 7 day neck pain was Org5, where neck pain for all participants increased by 1.0 at 12 months, but EET participants’ pain decreased by 1.7, indicating a significant increase in pain for EHP participants (the comparator).

During the intervention period, 112 participants discontinued their participation, primarily due to change of employer (*n* = 32) and excessive work demands preventing attendance at sessions (*n* = 26). Reasons for discontinuing by allocation, gender and organisation are presented in the Additional file 1: Table S3. During the intervention period, more EET participants (12.0%) discontinued than EHP participants (8.9%); more females (9.0%) discontinued than males (6.7%); and discontinuation rates varied widely across organisations (7.0, 27.1%), although these changes were not statistically significant.

At week 12, the most common reasons for not participating in training in the preceding four weeks (multiple options could be selected; 583 responses in total given) were: lack of time (*n* = 134), illness (*n* = 30), and lack of motivation (*n* = 21). The most common reasons for not continuing healthier lifestyle changes (535 responses in total given) were: lack of time (*n* = 144), lack of motivation (*n* = 121), and illness (*n* = 33).

When asked during the week 12 survey what they did or did not like about the programs, several EET participants noted that participating in the exercise training sessions had changed their thinking about strengthening exercises, particularly for the neck, and that they would be more likely to think positively about similar exercises in the future. A number of EET participants stated that they felt stronger and could see improvements in their neck pain, which motivated them to continue attending. Other comments provided were: *“(it) created networks with colleagues from across the branch that I wouldn’t otherwise get to know,”* and *“I met people new people on my floor and we rallied around each other in support”* (female participants, Org15). However, several participants noted that they found it difficult to fit participation into their work day, or that they were uncomfortable performing the neck exercises, as it was something they had not done before or they found some of the equipment (head gear utilised for neck exercises) uncomfortable to wear.

### Adoption

The research team invited 21 organisations to participate in the study (11 public sector, seven private sector, two government-operated businesses and one university). Of these, 14 accepted (66.6%) and seven declined (five due to planned organisational restructuring; one did not have the resources available to coordinate their participation; one provided no reason for non-involvement). Of the 14 organisations that participated, eight were from the public sector (local, state or federal government); four were private organisations; one was a university and one a government-operated business. The public and private sector organisations that declined participation were equivalent to participating organisations in relation to their organisation size, the size of the proposed recruitment pool and the co-location of administrative staff.

When asked during the interview why their organisation participated in this study, key themes included that they were actively looking for activities for their desk-based staff that would complement in-house wellness programs (e.g. *“it will give our workers a good opportunity to participate in a program that we know has some evidence behind it”* and “*we were looking at ways … of getting a healthier workplace”* (liaison, org 14)) and that they were looking to participate in and support research (e.g. *“this will be a little bit different. We’ll probably learn a lot from it*” and “*we certainly thought that this would be … an opportunity to look at … what other research activities are happening out there and also be part of that process”*(liaison, org 7))*.*

Four types of intervention agents were utilised for this study: physiotherapists to deliver the exercise training intervention; health professionals to deliver the health promotion intervention; physiotherapists and occupational therapists to conduct workstation assessments; and a research manager to coordinate recruitment, intervention and assessment activities. The session facilitators (*n* = 5) who delivered the interventions were approached directly through the contact network of the research team, due to their experience delivering interventions and assessments in office settings; no one approached declined involvement. The research manager (*n* = 1) was recruited through a formal recruitment method that attracted 13 candidates, 10 of whom were excluded due to lack of expertise.

### Implementation – organisation perspective

Participating organisations were required to coordinate communication activities in accordance with the study’s communication strategy; provide a consistent, suitable space for implementation activities; and appoint an onsite liaison to coordinate research activities. Compliance with these requirements is reported in Table 2 and varied between organisations. Not all organisations provided a consistent, suitable space for the conduct of research activities. To account for these variations, organisations were placed into three categories: consistent venue (no room changes), some room changes (fewer than 8 changes), and frequent changes (8 or more changes). The variations in seniority of the nominated onsite liaison were classified as junior administrative staff or external contractor, mid-level officer, or manager/senior official.

**Table 2:** Information on participating organisations – size, recruitment and organisational compliance

	Industry	Org size - employees (‘000)	Recruitment pool size	Allocated to intervention (% of pool)	Allocated (n) (EET; EHP)	Liaison seniority	Venue changes	Incentives offered
Org1	Public	< 1	557	12.2	57 (29; 28)	External contractor	Consistent	No
Org2	Private	1 - < 5	380	25.8	75 (36; 39)	Junior officer	Some	No
Org3	Public	< 1	64	76.6	44 (20; 24)	Mid-level officer	Consistent	No
Org4	Public	< 1	308	19.8	53 (23; 30)	Mid-level officer	Consistent	No
Org5	Tertiary education	< 1	207	21.8	37 (18; 19)	Mid-level officer	Some	No
Org6	Public	> 10	194	27.3	48 (26; 22)	Mid-level officer	Consistent	No
Org7	Public	> 10	702	16.5	99 (52; 47)	Mid-level officer	Frequent	No
Org8	Public	5–10	332	29.5	81 (42; 39)	Mid-level officer	Some	No
Org9	Private	> 10	116	49.1	50 (25; 25)	Manager or senior official	Consistent	No
Org10	Public	1 - < 5	195	41.0	68 (33; 35)	Manager or senior official	Consistent	No
Org11	Public	5–10	161	32.3	35 (18; 17)	Mid-level officer	Consistent	Yes
Org12	Private	< 1	459	9.4	35 (16; 19)	Mid-level officer	Some	Yes
Org13	Public	5–10	300	16.0	39 (20; 19)	Mid-level officer	Frequent	Yes
Org14	Private	> 10	54	83.3	42 (23; 19)	Manager or senior official	Consistent	Yes
All Orgs			4029	22.7	763 (381; 382)			

Feedback provided in the week 12 survey identified session scheduling as both a positive aspect (e.g. “*Not doing it on my own. Time was set aside and management support to participate*” female participant, Org1), and a barrier to adherence (e.g. “*allow people to choose their most suitable time and commit to that time. My group would arrive to do our exercise and there wouldn’t be enough space because others decide to go whenever they felt like it*” (female participant, Org9)).

### Implementation – participant perspective

Results for adherence to EET and EHP across all 14 organisations during the intervention and maintenance periods are reported in Table 3. Adherence rates varied considerably across organisations. Mean predicted adherence to EET sessions by organisation ranged from 40.9% (Org4) to 71.2% (Org9), with an overall mean of 55.7%. Mean observed adherence to EHP sessions by organisation raged from 28.2% (Org12) to 77.3% (Org9), with an overall mean of 56.2%.

**Table 3:** Organisational variations by RE-AIM domain

	Allocated (n) (EET; EHP)	Mean EET Adherence (intervention period^a^) (12 months^b^) (%)	Mean EHP Adherence (intervention period^a^) (12 months^b^) (%)	n	Productivity loss^c^ (baseline to 12 weeks; baseline to 12 months) (EET^e^)	n	Neck pain^d^ (baseline to 12 weeks) (EET^e^)	Attrition (12 weeks; 12 months) (%)
Org4	53 (23; 30)	40.9 33.3	49.4 81.8	35 19	0.1 (EET 0.4) −0.2 (EET 0.4)	35 18	−0.5 (EET 0.4) −0.2 (EET 0.4)	22.6; 43.4
Org13	39 (20; 19)	57.7 25.0	53.4 54.5	33 24	0.2 (EET − 0.2) − 0.1 (EET − 0.2)	33 24	− 0.2 (EET 0.0) 0.1 (EET − 0.5**)	10.3; 10.3
Org9	50 (25; 25)	71.2 12.5	77.3 70.6	43 34	− 0.2 (EET 0.3) − 0.1 (EET 0.2)	44 34	− 0.7 (EET 0.4) − 0.7 (EET 1.1)	8.0; 14.0
Org5	37 (18; 19)	61.2 0.0	66.2 61.5	33 26	0.0 (EET 0.0) 0.0 (EET 0.0)	33 25	0.2**(EET −0.3) 1.0** (EET −1.7**)	13.5; 18.9
Org14	42 (23; 19)	67.1 7.7	45.0 62.0	32 19	0.1 (EET −0.1) 0.0 (EET 0.0)	32 19	−0.2 (EET − 0.2) −1.0 (EET 0.9)	9.5; 19.0
Org12	35 (16; 19)	54.4 0.0	28.2 100.0	19 9	0.0 (EET 0.1) 0.0 (EET 0.3)	19 9	0.9** (EET −1.4**) 0.3 (EET − 1.1)	20.0; 51.4
Org1	57 (29; 28)	48.0 36.4	53.2 52.9	46 29	0.1 (EET 0.0) 0.1 (EET −0.3)	46 28	−1.0** (EET 0.2) − 1.0 (EET 0.8)	7.0; 22.8
Org7	99 (52; 47)	62.8 8.0	51.2 64.0	70 52	0.0 (EET −0.3) 0.1 (EET − 0.3)	70 51	− 0.4 (EET 0.0) 0.1 (EET − 0.1)	17.2; 24.2
Org6	48 (26; 22)	47.8 12.5	54.6 45.5	33 19	0.0 (EET −0.1) 0.1 (EET − 0.2)	33 19	− 0.8* (EET 0.6) − 0.6 (EET 0.1)	27.1; 37.5
Org10	68 (33; 35)	54.4 11.8	59.3 54.5	52 39	0.1 (EET 0.1) 0.2 (EET −0.2)	51 39	0.0 (EET 0.1) 0.4 (EET −0.1)	8.8; 17.6
Org8	81 (42; 39)	46.4 13.3	53.1 52.2	62 38	−0.1 (EET 0.0) 0.2 (EET 0.0)	64 38	−0.5 (EET 0.5) − 0.1 (EET 0.2)	19.8; 28.4
Org11	35 (18; 17)	59.8 37.5	55.9 66.7	29 20	0.4* (EET −0.3) 0.3 (EET − 0.4)	29 20	− 0.1 (EET − 0.4) − 0.4 (EET 0.6)	14.3; 20.0
Org3	44 (20; 24)	55.9 11.1	61.1 50.0	35 20	0.4* (EET −0.1) 0.5 (EET − 0.2)	35 19	0.0 (EET − 0.1) − 0.4 (EET − 0.1)	18.2; 25.0
Org2	75 (36; 39)	54.1 18.8	68.8 65.2	66 40	0.4* (EET −0.1) 0.5** (EET − 0.2)	66 40	− 0.1 (EET − 0.6) − 0.5 (EET 0.0)	9.3; 24.0
All Orgs	763 (381; 382)	55.7 15.0	56.2 62.0	588 366	0.1* (EET 0.0) 0.2**(EET −0.1*)	383	−0.3* (EET 0.0) − 0.2 (EET 0.0)	14.7; 25.2

^a^data collected upon completion of the 12 week intervention period

^b^data collected at 12 months post-commencement

^c^cost in days of health-related productivity loss

^d^neck pain past 7 days

^e^coefficient for interaction of EET allocation over time

* *p* ≤ 0.05

** *p* ≤ 0.001

Adherence levels at 12 months are reported by organisation in Table 3**.** Mean reported regular adherence to EET by organisation raged from 0.0% (Org5 and Org12) to 37.5% (Org11), with an overall mean of 15.0%. Mean reported regular adherence to EHP by organisation raged from 45.5% (Org6) to 100.0% (Org12), with an overall mean of 62.0%, though it should be noted that ‘maintaining healthier lifestyle changes’ requires a lower time commitment than exercising three times per week. The most commonly reported reasons for not training (totalled from month 4 to month 12) were lack of time (33.8%, *n* = 602/1777 responses) and lack of motivation (28.6%, *n* = 508/1777 responses). The most commonly reported reasons for not practising changes were lack of time (33.8%, *n* = 750/2219 responses), lack of motivation (26.7%, *n* = 593/2219 responses) and hard to start after illness or vacation (12.7% 281/2219 responses).

During the 12 week intervention period, 14.7% of participants discontinued their participation without formally withdrawing from the study (Table 3). At the end of the 12 month period, 25.2% had formally discontinued. Attrition rates varied widely between organisations, ranging from 7.0% (Org1) to 27.1% (Org6) at the end of 12 weeks; and from 10.3% (Org13) to 43.4% (Org4) at the end of 12 months. However, incomplete data from participants still enrolled in the study was of concern, with complete data sets (survey and strength measures) received from 69.1% (*n* = 527) of the original 763 participants at 12 weeks; and 40.8% (*n* = 311) at 12 months.

The total time commitment for each participant was approximately 16 h (12 h for attendance at EET or EHP sessions, one hour for workstation assessments, and three hours for research-specific components such as surveys, physical assessments and exercise diary completion).

### Implementation – research perspective

#### Protocol variations

The study was implemented largely as intended. This cluster-randomisation process resulted in the balanced allocation of individuals to each intervention (*n* = 381 for EET and *n* = 382 for EHP) and produced two groups of participants that only differed slightly in relation to health-related quality of life, number of medical conditions and workstation standards [10]. However, there were some variations from the original study protocol relating to: cluster allocation and sample size; scheduling of EET sessions; and introduction of incentives.

The cluster size in the study protocol was originally identified as five to eight participants [9]. Whilst mean cluster size was within that range (7.6), in some cases, organisational structures and the work location of teams (e.g. open plan offices), necessitated the formation of larger or smaller clusters (range 3–17) to ensure homogeneity within and heterogeneity between clusters and to reduce the risk of contamination between intervention arms. Additionally, the trial was initially powered for a sample size of 640 participants. However, organisational restructuring during 2013 and 2014 created higher than anticipated loss to follow-up at 12 weeks (14.7% compared to a predicted 10%, with no complete clusters lost). Consequently, the desired sample size was increased to 720 in 2014, with 763 participants across 100 clusters finally recruited and allocated to an intervention arm (an extra 43 participants were recruited due to higher than expected uptake in the final two organisations).

Scheduling of EET sessions varied from the study protocol to include an additional supervised training session in the first week to allow sufficient time for participants to be trained on safe and effective performance of exercises and completion of the exercise diaries. Additionally, the schedule was impacted by public holidays, school holidays, local business needs and the availability of suitable venues. This affected both interventions and most organisations.

Incentives for higher adherence to sessions during the intervention period were introduced after the first 10 organisations, prompted by lower than anticipated adherence rates over the 12-week intervention period. Two levels of incentives were offered to 267 participants – resources with an approximate value of 40AUD for attending 65 to 90% of sessions; and resources with an approximate value of 100AUD for attending more than 90% of sessions. Higher level incentives were awarded to 16% of potentially eligible participants overall (23% of EET participants (16 female, 14 male), 10% of EHP participants (8 female, 5 male)); and lower level incentives were awarded to 22% of potentially eligible participants overall (25% of EET participants (20 female, 12 male), 20% of EHP participants (15 female, 12 male)). Mean observed adherence for pre-incentive EET participants was 7.3 sessions (SD 3.7) and 8.1 (SD 3.3) sessions once incentives were introduced (*p* = 0.055), while mean EHP sessions attended pre-incentives was 6.8 (SD 3.5) and 6.6 (SD 3.3) once incentives were offered (*p* = 0.694).

#### Consistency

The implementation was staffed consistently across all organisations and intervention components. Workstation assessments were delivered by five trained health professionals (488; 203; 38; 16; and two assessments each). In total, 884 supervised exercise training sessions were delivered (to 68 exercise groups across 14 organisations). Of these, all but six were delivered by a single physiotherapist. A researcher directly involved in the design of the EET protocol (SOL) randomly audited a day of training sessions in six organisations to ensure consistency of program delivery with the guidelines and study protocol and safety of participants. Two experienced health professionals were engaged to develop and deliver the EHP sessions. With two exceptions, the same facilitator delivered EHP sessions at each site to ensure continuity and build rapport and trust between the facilitator and the group. The health professionals met regularly to ensure consistency in delivery style.

#### Implementation costs

Equipment was purchased for 14.7% of participants (compared to a predicted 20%), based on their workstation assessments, and small items were sourced in the workplace and allocated for the use of a further 10.4% of participants. Education on the functionality and safe use of equipment already onsite enabled workstations to be adapted to meet the needs of the majority of participants. Consequently, funds expended on office equipment were lower than anticipated (17,365AUD expended; 25,600AUD budgeted).

### Maintenance – setting level

None of the 14 participating organisations took steps to continue the exercise training or health promotion interventions beyond the study period. Participants in one organisation formed a working group to canvas ideas for improving their health and wellbeing. The research team were invited to contribute to this group.

During the liaison interviews with four organisations, common themes on not adopting the intervention included the work required to maintain the program, the lack of meeting space, and changes of leadership within the organisation. One liaison noted, however, that participating in the study had increased participation in other wellness activities *‘and there’s getting more take-up of these types of activities, so if you provide the right environment … ’* (liaison, Org14).

## Discussion

This paper examined the implementation of a workplace study using the RE-AIM framework domains of reach, effectiveness, adoption, implementation and maintenance. The interventions were evaluated in the context of a cluster-randomised effectiveness trial with the research team closely involved in recruitment, organisation and delivery of the EET and EHP sessions. Despite this approach, there were considerable variations between all organisations for each of the RE-AIM dimensions, including effectiveness. These organisational variances were examined to understand their potential impact on outcomes.

Overall reach was lower than anticipated (at 18.9%), although consistent with previous similar workplace-based studies [25–27]. Reach varied widely across organisations. The higher recruitment rates (e.g. > 45%) were from private and public organisations with relatively small recruitment pools (range 54 to 116). In contrast, organisations with lower recruitment rates (e.g. < 20%) drew participants from larger recruitment pools (range 459 to 702). This relationship has been demonstrated in previous examinations of recruitment predictors for workplace exercise interventions [25]. The size of the recruitment pool, however, did not always reflect the size of the organisation, with some organisations offering participation to only selected units or branches. These findings suggest that, rather than maximising the size of the recruitment pool within organisations in the hope of recruiting sufficient participants, larger organisations should be encouraged to ‘stagger’ recruitment and target smaller teams and work units. This approach may also enable team leaders to engage more directly with potential participants.

Implementation effectiveness in relation to the primary outcome of health-related productivity varied across organisations, with no apparent pattern between positive or negative changes and other RE-AIM dimensions. For instance, Org4 reported very low intervention adherence and the highest attrition rate at 12 months, yet also recorded the highest reduction in health-related productivity loss at 12 months across all participants, while recording an increase in both health-related productivity loss and pain for EET participants. This result may be partially explained by the lower baseline productivity costs of participants who submitted 12 month data, as those with higher costs did not have data for inclusion in the 12 month analysis and no imputation of missing data was conducted.

Three organisations (Org9, Org10 and Org14) demonstrated strong commitment by appointing a senior liaison, providing a consistent venue for interventions and achieving high recruitment and (for Org9 and Org14) high intervention adherence. However, these organisations achieved different results. Org9 and Org14 achieved minor (0.1 days for Org9) or no reduction in productivity loss across all participants at 12 months (despite the significantly lower baseline costs of the participants who submitted data (1.1 days) in Org9) and a decrease in neck pain across all participants at 12 months (− 0.7 for Org9 and − 1.0 for Org14), while EET participants recorded an increase in neck pain in both organisations (1.1 for Org9 and 0.9 for Org14) and an increase in productivity costs (0.2 days) in Org9. The results in Org10 were the reverse, with increases in pain and productivity at 12 months for all participants, but reductions for EET participants.

Organisational participation in this study required a commitment of considerable resources, including staff time and provision of session space. In return, participating staff received a workstation assessment (with associated equipment) and a 12 week health intervention. The high adoption rate among public and private organisations reflected the awareness among employers of the impact of neck pain on office workers and their willingness to try innovative approaches to improve the health and wellbeing of their staff. However, once organisations had agreed to participate, they demonstrated different levels of commitment in terms of resource allocation (liaison seniority, venue changes, and communications) and their capacity to engage and encourage staff to participate, with mixed effects on reach and implementation.

The seniority of staff engaged in the intervention also varied between organisations. With one exception, the organisations that nominated managers or senior officers as liaisons had higher recruitment rates than those with more junior liaisons. Conversely, the percentage of participants in management positions (where managers joined the study and led by example), did not have a direct effect on either recruitment or adherence during the intervention period. For instance, Org 9, who appointed a senior-level liaison and achieved both high reach and high intervention adherence, only had 4% of participants in management positions, while Org14 had 33% of participants in management positions and also achieved very high reach and high EET intervention adherence.

The primary and secondary outcomes by organisation reported here should be interpreted with caution. The study was powered for analysis at the whole sample level and loss to follow-up at 12 months of over 25% in five organisations and a pool of fewer than 20 participants in four organisations means results can only be treated as indicative. The organisation with the most significant results (Org2), showed an increase in health-related productivity loss (a higher cost to the organisation) across all participants at both 12 weeks and 12 months, but potential reductions in health-related productivity loss for EET participants. The three organisations (Org9, Org10 and Org14) with the strongest cumulative organisational commitment (senior liaison, consistent venue and regular communication) achieved high reach, generally high adherence, and low attrition at 12 months, but did not demonstrate consistent results, with both increases and decreases in productivity loss.

There was also an apparent disconnect in six organisations between changes in productivity loss and changes in neck pain. Five organisations (Org1, Org2, Org3, Org11 and Org14) reported either no change or an increase in productivity loss, while also reporting considerable reductions in neck pain. Conversely, Org5 reported no change in productivity loss, but an increase in neck pain for all participants of 1.0 and a decrease for EET participants of 1.7. The mixed results reported here led us to consider other factors that may have been a source of variation and that a change in health may not always be reflected in a change in productivity outcomes.

The EET intervention included in the study [9] was modified from one delivered to office workers in Denmark [28]. The Danish intervention was conducted for one hour per week during work hours across 12 offices of a single, large public organisation, for an intervention period of 12 months. The study compared specific resistance training and all-round physical exercise to a reference group and found that both exercise interventions effectively reduced neck pain. The inclusion of the productivity analysis in the study provides further insight into the changes that exercise interventions can affect in organisations. For instance, that reductions in neck pain are not always associated with productivity improvements and vice versa.

A potential limitation of this study was the lack of information collected on organisational culture with no information collected on leadership (including leading by example [29] or organisational commitment to employee wellbeing and health [30]). Consequently, our ability to assess organisational variances was limited to observed factors. It should also be noted that no steps were taken to isolate the impact of the workstation assessment before exercise training or health promotion sessions commenced, and that the EHP intervention may have had a ‘placebo’ effect that could not be evaluated. Other limitations included the lack of a true control group, incorporation of a workstation assessment in both interventions, the impact of busy workplaces on interventions, and the limited opportunities for influencing participant behaviours. Future studies should aim to include a true control group to clarify the impact of the combined ergonomic and exercise intervention.

All participants received an individual workstation assessment prior to allocation to EET or EHP. Although evidence for the effectiveness of ergonomic interventions for neck pain is limited [31–34], it is possible that changes to workstations reduced neck pain for participants in both intervention arms. Further, the workstation assessments included recommendations for taking regular breaks and the re-arrangement of desktop equipment to suit the participant’s work flow, which may have affected self-rated perceptions of productivity. However, the standard of workstations assessed was high (mean 86% (31.6 out of 38)), leaving minimal room for improvement.

Although the study was implemented as pragmatically as possible, work demands often precluded participants from attending every week, and staff turnover contributed to attrition during the intervention period. This was evidenced by ‘excessive work demands’ being the second-most cited reason for discontinuing participation and ‘lack of time’ being the most frequently cited reason for absence from training during the maintenance period. While some participants suggested offering the exercise and health promotion activities outside of work hours, many employees considered delivery during work hours to be a desirable feature of the interventions. Improving the flexibility of session availability could be considered for future studies.

The intervention protocol allowed limited opportunities to directly influence adherence to EET and EHP sessions. No behaviour modification strategies were included in the exercise protocol and the health promotion program included only one session on goal-setting with no monitoring of health improvement goals. Such factors have been identified as key for promoting and maintaining behaviour change, particularly in sedentary adults [35]. Furthermore, the use of paper diaries that were submitted at the completion of the intervention period to monitor adherence provided little opportunity to track and motivate individual participants. Future interventions should include better participant activity tracking (e.g. online diaries) and behaviour modification approaches (such as tailored health coaching) to increase intervention adherence.

In summary, future interventions designed to assess the impact of workplace-based exercise training for office workers should stagger recruitment activities where possible to draw from smaller participant pools (50–100 employees); clearly articulate study requirements to organisations interested in participating; improve the flexibility of session availability where possible; include behaviour modification strategies with electronic/real-time activity tracking; adjust for potential ‘placebo’ effects where there is an active comparison intervention, or use a non-intervention control group.

## Conclusions

The study showed that the workplace-based combined intervention for office workers that included best practice ergonomics and strength-based exercise training did result in lower health-related productivity loss than those that include ergonomics and health promotion information. However, both combined interventions reduced neck pain in office workers. The process evaluation presented here showed that, although the study protocol was implemented with high consistency and fidelity, variations in four domains (adoption, reach, implementation and effectiveness) arose between the 14 participating organisations. These variations may be the source of mixed effectiveness across organisations, but sufficient data was not collected for an obvious pattern to emerge. Factors known to increase the success of workplace interventions, such as strong management support, a visible commitment to employee wellbeing and participant engagement in intervention design should be considered and adequately measured for future interventions.

## Supplementary information

**Additional file 1: Table S1.** Reach and representativeness across 14 organisations (adjusted income) **Table S2.** Comparison of baseline responses of participants with and without 12 week or 12 month data. **Table S3.** Reasons for discontinuation during implementation and maintenance periods

## Abbreviations

EET: Ergonomic and Exercise Training
EHP: Ergonomic and Health Promotion
Org: Organisation
SD: Standard Deviation
