# Supplementary material for: Process evaluation of a workplace-based health promotion and exercise cluster-randomised trial to increase productivity and reduce neck pain in office workers: a RE-AIM approach
Source: BMC Public Health. 2020 Feb 4;20:180. doi: 10.1186/s12889-020-8208-9 (PMC7001341; doi:10.1186/s12889-020-8208-9)
Supplement: Supplementary file 1 — Additional file 1: Table S1. Reach and representativeness across 14 organisations (adjusted income) Table S2. Comparison of baseline responses of participants with and without 12 week or 12 month data. Table S3. Reasons for discontinuation during implementation and maintenance periods [file 12889_2020_8208_MOESM1_ESM.docx]

## Supplement

#### Table S.1: Reach and representativeness across 14 organisations (adjusted income)

| Organisation | Pool | Number registering interest | Reach (%) | **Recruitment pool** | | | | | **Allocated participants** | | | | |
| --- | --- | --- | --- | --- | --- | --- | --- | --- | --- | --- | --- | --- | --- |
|  |  |  |  | Age in years - mean | | Gender  (% female) | Gross annual income AUD ‘000 (mean, SD)^#^ | | Age in years - mean | | Gender  (% female) | Gross annual income AUD ‘000 (mean, SD) ^#^ | |
|  |  |  |  | Male | Female |  | Male | Female | Male | Female |  | Male | Female |
| Org 1  (public) | 557 | 68 | 12.2 | - | - | 57.9 | 69,767  (7,355) | | 46.7 | 45.5 | 66.7 | 72.4 (10.5) | 66.2 (10.4) |
| Org 2  (private) | 380 | 98 | 25.8 | 41.5 | 40.8 | 23.1 | 100.8 (8.4) | 70.5  (3.7) | 40.7 | 39.5 | 26.7 | 102.7 (27.1) | 70.4 (24.4) |
| Org 3  (public) | 64 | 49 | 76.6 | - | - | 76.6 | 74.9 (16.0) | 74.8  (15.9) | 35.2 | 39.6 | 83.3 | 78.4 (18.6) | 77.6 (14.4) |
| Org 4  (public) | 308 | 61 | 19.8 | 44.0 | 40.0 | 60.7 | 78.9  (6.1) | 66.6  (4.1) | 46.1 | 43.1 | 61.7 | 79.0 (23.6) | 73.9 (17.8) |
| Org 5  (private) | 207 | 45 | 21.8 | 43.0 | 52.0 | 72.0 | 66.4  (5.5) | 67.7  (6.5) | 38.5 | 44.2 | 83.8 | 73.1 (15.1) | 61.9 (11.0) |
| Org 6  (public) | 194 | 53 | 27.3 | 42.4 | 42.1 | 52.1 | 87.0  (5.5) | 75.2  (5.0) | 44.4 | 39.4 | 79.6 | 88.0 (23.0) | 79.3 (19.9) |
| Org 7  (public) | 702 | 116 | 16.5 | 47.5 | 41.5 | 68.0 | 99.6  (10.2) | 88.8  (9.1) | 43.5 | 44.7 | 74.5 | 106.3 (29.7) | 86.7 (19.2) |
| Org 8  (public) | 332 | 98 | 29.5 | 48.4 | 37.3 | 53.9 | 94.8  (9.9) | 88.7  (9.6) | 44.4 | 39.4 | 55.1 | 91.2 (16.8) | 85.1 (31.0) |
| Org 9  (private) | 116 | 57 | 49.1 | 42.3 | 43.3 | 59.5 | - | - | 39.9 | 42.5 | 64.0 | 82.0 (25.2) | 67.8 (18.5) |
| Org 10  (public) | 195 | 80 | 41.0 | - | - | 41.5 | - | - | 43.6 | 44.1 | 53.7 | 77.8 (18.4) | 71.3 (14.9) |
| Org 11  (public) | 161 | 52 | 32.3 | 45.0 | 43.0 | 53.4 | - | - | 48.5 | 46.4 | 64.7 | 75.0 (8.1) | 81.4 (16.8) |
| Org 12  (private) | 459 | 43 | 9.4 | 42.0 | 39.0 | 30.7 | 133.1  (26.7) | 110.8  (11.0) | 41.8 | 42.0 | 45.5 | 148.4 (64.8) | 127.9 (58.9) |
| Org 13  (public) | 300 | 48 | 16.0 | - | - | - | - | - | 40.2 | 43.0 | 59.0 | 98.3 (21.2) | 91.2 (15.5) |
| Org 14  (private) | 54 | 45 | 83.3 | 43.0 | 34.0 | 14.8 | 143.9  (33.0) | 122.5  (16.9) | 42.0 | 35.5 | 47.5 | 213.1. (102.8) | 114.2 (45.0) |
| All Orgs | 4029 | 913 | 22.7 | 43.1* | 41.8* | 48.9 | 101.9  (8.6) | 79.9  (3.6) | 42.8 | 42.6 | 61.0 | 99.2 (50.1) | 79.7 (26.6) |

* weighted average age across all organisations based on data provided; income adjusted to June 2015

Note: All requested data were received from eight organisations and partial data were received from the remaining six.

#### Table S.2: Comparison of baseline responses of participants with and without 12 week or 12 month data.

|  | Productivity^a^ | | | | | Neck pain^b^ | | | | |
| --- | --- | --- | --- | --- | --- | --- | --- | --- | --- | --- |
| Org | Complete (mean) (12w; 12m) | n | Incomplete (mean) (12w; 12m) | n | Diff | Complete (mean) (12w; 12m) | n | Incomplete (mean) (12w; 12m) | n | Diff |
| Org1 | 4.4 | 46 | 3.5 | 8 | -0.9 | 1.7 | 46 | 2.5 | 8 | 0.8 |
|  | 3.7 | 29 | 4.9 | 25 | 1.2* | 1.4 | 28 | 2.2 | 26 | 0.7 |
| Org2 | 3.0 | 66 | 2.4 | 8 | -0.6 | 1.6 | 66 | 1.3 | 9 | -0.3 |
|  | 2.7 | 40 | 3.2 | 34 | 0.5 | 1.5 | 40 | 1.7 | 35 | 0.2 |
| Org3 | 4.0 | 35 | 3.8 | 7 | -0.2 | 1.3 | 35 | 2.1 | 7 | 0.8 |
|  | 4.0 | 20 | 4.0 | 22 | 0.1 | 1.3 | 19 | 1.6 | 23 | 0.3 |
| Org4 | 4.0 | 35 | 2.9 | 12 | -1.1* | 1.3 | 35 | 1.3 | 12 | -0.1 |
|  | 4.2 | 19 | 3.4 | 28 | -0.7 | 1.2 | 18 | 1.4 | 29 | 0.2 |
| Org5 | 4.4 | 32 | 4.8 | 5 | 0.4 | 1.2 | 33 | 0.3 | 4 | -1.0 |
|  | 4.4 | 26 | 4.6 | 10 | 0.2 | 1.2 | 25 | 0.8 | 12 | -0.4 |
| Org6 | 4.8 | 33 | 3.3 | 15 | -1.6* | 1.9 | 33 | 2.7 | 15 | 0.8 |
|  | 5.0 | 19 | 3.9 | 29 | -1.2* | 2.1 | 19 | 2.2 | 29 | 0.2 |
| Org7 | 4.6 | 70 | 4.1 | 27 | -0.5 | 1.5 | 70 | 1.8 | 27 | 0.2 |
|  | 4.4 | 52 | 4.4 | 45 | 0.1 | 1.7 | 51 | 1.5 | 46 | -0.1 |
| Org8 | 3.9 | 38 | 5.3 | 40 | 1.3* | 1.5 | 64 | 1.3 | 14 | -0.3 |
|  | 4.0 | 62 | 4.4 | 16 | 0.3 | 1.4 | 38 | 1.5 | 40 | 0.1 |
| Org9 | 3.3 | 43 | 4.5 | 7 | 1.2 | 1.8 | 44 | 1.7 | 6 | -0.1 |
|  | 3.0 | 34 | 4.2 | 16 | 1.1* | 1.7 | 34 | 1.9 | 16 | 0.2 |
| Org10 | 4.2 | 52 | 4.4 | 15 | 0.2 | 1.7 | 51 | 1.0 | 16 | -0.7 |
|  | 4.2 | 29 | 4.2 | 28 | 0.0 | 1.7 | 39 | 1.4 | 28 | -0.2 |
| Org11 | 3.6 | 29 | 4.9 | 5 | 1.3 | 1.2 | 29 | 2.4 | 5 | 1.2 |
|  | 3.6 | 20 | 4.1 | 14 | 0.6 | 1.6 | 20 | 1.1 | 14 | -0.4 |
| Org12 | 3.3 | 19 | 3.5 | 14 | 0.2 | 1.5 | 19 | 1.1 | 14 | -0.5 |
|  | 3.9 | 9 | 3.2 | 24 | -0.7 | 0.7 | 9 | 1.6 | 24 | 0.9 |
| Org13 | 4.6 | 33 | 5.0 | 6 | 0.3 | 2.1 | 33 | 0.3 | 6 | -1.7 |
|  | 4.8 | 24 | 4.4 | 15 | -0.4 | 1.6 | 24 | 2.1 | 15 | 0.6 |
| Org14 | 4.9 | 32 | 2.9 | 8 | -2.0* | 1.8 | 32 | 3.9 | 8 | 2.1* |
|  | 4.2 | 19 | 4.8 | 21 | 0.6 | 1.8 | 19 | 2.6 | 21 | 0.8 |
| All orgs | 4.1 | 587 | 3.9 | 152 | -0.1 | 1.6 | 590 | 1.7 | 151 | 0.1 |
|  | 4.0 | 388 | 4.1 | 351 | 0.1 | 1.5 | 383 | 1.7 | 358 | 0.2 |

^a^ cost in days of health-related productivity loss

^b^ neck pain past 7 days

* p≤0.05

#### Table S.3: Reasons for discontinuation during implementation and maintenance periods

|  | **Intervention Period** | | | **Maintenance Period**  **(n (%))** | **Total for study period (n (%))** |
| --- | --- | --- | --- | --- | --- |
| **Reason** | **After allocation, before intervention (n)** | **During 12 week intervention (n)** | **Total prior to 12 week follow-up  (n (%))** |  |  |
| **By reason** | | | | | |
| not provided | 1 | 4 | 5 (0.65) | 9 (1.2%) | 14 (1.8%) |
| change of employer | 4 | 26 | 32 (4.2) | 60 (7.9%) | 92 (12.1%) |
| excessive work demands | 5 | 16 | 26 (3.4) | 6 (0.8%) | 32 (4.2%) |
| unrelated illness or injury | 2 | 8 | 11 (1.4) | 4 (0.5%) | 15 (2.0%) |
| excluded during baseline assessments | 6 | 0 | 27 (3.5) | 0 (0.0%) | 27 (3.5%) |
| pregnancy | 0 | 2 | 2 (0.3) | 0 (0.0%) | 2 (0.3%) |
| other | 4 | 2 | 9 (1.2) | 1 (0.1%) | 9 (1.2%) |
| Total | 22 | 58 | 112 (14.7) | 80 (10.5%) | 192 (25.2%) |
| **By organisation (industry sector)** | | | | | |
| Org 1 (public) | 1 | 2 | 4 (7.0) | 9 (15.8%) | 13 (22.8%) |
| Org 2 (private | 1 | 4 | 7 (9.3) | 11 (14.7%) | 18 (24.0%) |
| Org 3 (public) | 2 | 4 | 8 (18.2) | 3 (6.8%) | 11 (25.0%) |
| Org 4 (public) | 7 | 4 | 12 (22.6) | 11 (20.8%) | 23 (43.4%) |
| Org 5 (public) | 1 | 2 | 5 (13.5) | 2 (5.4%) | 7 (18.9%) |
| Org 6 (public) | 1 | 9 | 13 (27.1) | 5 (10.4%) | 18 (37.5%) |
| Org 7 (public) | 2 | 10 | 17 (17.2) | 7 (7.1%) | 24 (24.2%) |
| Org 8 (public) | 2 | 9 | 16 (19.8) | 7 (8.6%) | 23 (28.4%) |
| Org 9 (private | 0 | 3 | 4 (8.0) | 3 (6.0%) | 7 (14.0%) |
| Org 10 (public) | 0 | 1 | 6 (8.8) | 6 (8.8%) | 12 (17.6%) |
| Org 11 (public) | 2 | 1 | 5 (14.3) | 2 (5.7%) | 7 (20.0%) |
| Org 12 (private | 2 | 4 | 7 (20.0) | 11 (31.4%) | 18 (51.4%) |
| Org 13 (public) | 0 | 3 | 4 (10.3) | 0 (0%) | 4 (10.3%) |
| Org 14 (private | 1 | 2 | 4 (9.5) | 4 (9.5%) | 8 (19.0%) |
| **By gender** | | | | | |
| Male | 0* | 20 | 20 (6.7) | 32 (11.0%) | 52 (17.9%) |
| Female | 5* | 38 | 42 (9.0) | 48 (10.6%) | 92 (20.3%) |
| **By allocation** | | | | | |
| EET | 14 | 32 | 46 (12.1) | 37 (9.7%) | 83 (21.8%) |
| EHP | 8 | 26 | 34 (8.9) | 43 (11.3%) | 77 (20.2%) |

* 17 participants discontinued without completing the baseline survey
